# Effects of Supplementation with the Fat-Soluble Vitamins E and D on Fasting Flow-Mediated Vasodilation in Adults: A Meta-Analysis of Randomized Controlled Trials

**DOI:** 10.3390/nu7031728

**Published:** 2015-03-10

**Authors:** Peter J. Joris, Ronald P. Mensink

**Affiliations:** 1 Department of Human Biology, NUTRIM School of Nutrition and Translational Research in Metabolism, Maastricht University Medical Center, Maastricht 6200 MD, The Netherlands; E-Mail: r.mensink@maastrichtuniversisty.nl; 2 Top Institute of Food and Nutrition (TIFN), Wageningen 6709 PA, The Netherlands

**Keywords:** flow-mediated vasodilation, fat-soluble, vitamin D, vitamin E, meta-analysis

## Abstract

The effects of fat-soluble vitamin supplementation on cardiovascular disease (CVD) risk are not clear. Therefore, we performed a meta-analysis to quantify effects of fat-soluble vitamin supplements on fasting flow-mediated vasodilation (FMD) of the brachial artery, a validated marker to assess CVD risk. Randomized placebo-controlled trials (RCTs) were identified by a systematic search till July 2014. Seven RCTs studying the effects of vitamin E supplements (range: 300 to 1800 IU per day) and nine RCTs examining the effects of vitamin D supplements, that involved, respectively, 303 and 658 adults, were included. No studies with carotenoid or vitamin K supplements were found. Vitamin E supplementation increased FMD *vs*. control by 2.42% (95% CI: 0.46% to 4.37%; *p* = 0.015). No effects of vitamin D supplementation were found (0.15%; 95% CI: −0.21% to 0.51%; *p* = 0.41). These effects did not depend on subject characteristics, treatment characteristics or technical aspects of the FMD measurement. However, no dose-response relationship was evident for vitamin E, statistical significance depended on one study, while the levels of supplement were far above recommended intakes. The current meta-analysis, therefore, does not provide unambiguous evidence to support the use of fat-soluble vitamin supplements to improve fasting FMD in adults.

## 1. Introduction

Observational studies have found inverse associations between the use of fat-soluble vitamin supplements and cardiovascular disease (CVD) risk. However, data from intervention trials are in general disappointing, while for some of the fat-soluble vitamins hardly any data are available [1]. Alternatively, effects of these essential micronutrients on validated markers of CVD, such as vascular function markers can be studied [2]. Measurements of vascular endothelial function, which is a powerful predictor of atherosclerotic disease progression and cardiovascular event rates [3], may indeed be useful to demonstrate CVD benefits [4]. Unfortunately, results of studies on the effects of fat-soluble vitamin supplementation on flow-mediated vasodilation (FMD) of the brachial artery, which is considered as the non-invasive gold standard technique to assess vascular endothelial function [5], were not consistent.

Several papers have therefore summarized the results for vitamin E and vitamin D. Montero and colleagues concluded that longer-term antioxidant vitamin supplementation could improve endothelial function in non-obese adults [6], but (i) only studies involving subjects with type II diabetes were included and (ii) effects of vitamin E could not be untangled from the known positive relationship between vitamin C and endothelial function [7]. Further, Min [8] and Liu *et al.* [9] reviewed clinical studies focusing on vitamin D supplementation and endothelial function, but results were equivocal. Also, quantitative estimates of effect sizes were lacking [8,9] and results of several relevant clinical trials were not included [8]. We therefore performed a meta-analysis of randomized placebo-controlled trials (RCTs) on the effects of fat-soluble vitamin supplementation on fasting FMD in adults. Further, the impact of (i) subject characteristics; (ii) treatment characteristics; and (iii) technical aspects of the FMD measurement on the effects observed on FMD was examined.

## 2. Experimental Section

The present meta-analysis was reported according to the Preferred Reporting Items for Systematic reviews and Meta-Analyses (PRISMA) guidelines [10].

### 2.1. Search Strategy

Potentially relevant studies were identified by a systematic search of Medline, Embase and the Cochrane Library database (Cochrane Central Register of Clinical Trials) till July 2014. The following search terms were used to search in titles and abstracts: (vitamin or supplement or calciferol or tocopherol or tocotrienol or retinol or carotenoid or carotene) and (flow mediated vasodilation (or vasodilatation or dilation or dilatation) or endothelial (or endothelium) dependent vasodilation (or vasodilatation or dilation or dilatation) or endothelial (or endothelium) function (or dysfunction) or FMD or vascular reactivity or brachial artery). The search was limited to studies in humans and to the English language. Reference lists from the selected articles were also screened manually for potentially relevant publications.

### 2.2. Selection of Trials

Randomized placebo-controlled trials, which investigated the relationship between fat-soluble vitamin supplementation and fasting FMD of the brachial artery with parallel and crossover designs, were selected.

The selection was performed in two steps. First, titles and abstracts were screened. Studies were selected if they met the following inclusion criteria: human intervention study with adults, intervention with fat-soluble vitamin supplements as experimental variable, no intentional co-intervention that made it impossible to estimate the effect of fat-soluble vitamin supplementation, and assessment of fasting vascular endothelial function by measuring FMD. In the second step, full-texts of the selected articles were read and studies were excluded based on the following criteria: no full text available (conference abstracts), missing data on FMD, no appropriate measures of variability reported, intentional co-intervention and no suitable placebo control treatment (prospective cohort studies). Both authors (Peter J. Joris and Ronald P. Mensink) completed the systematic literature search independently. When inconclusive, eligibility was discussed until consensus was reached.

### 2.3. Data Extraction

For each of the selected studies, data were extracted using a custom-made database including identification of the study (first author’s name and year of publication), study design (parallel or crossover), subject characteristics (sample size, age, gender, body mass index (BMI), baseline FMD level, and health status), treatment characteristics (type of vitamin supplements, total supplement dose and duration of follow-up), technical aspects of the FMD measurement (position of cuff and time of occlusion) and FMD values with accompanying measures of variance. For vitamin E, doses in mg were transformed into international units (IU) [11].

### 2.4. Statistical Analysis

Statistical analyses were performed using Stata 12.1 software (Stata Corporation, College Station, TX, USA). The FMD response was quantified as the maximal percentage change in post occlusion arterial diameter relative to baseline diameter, which is the diameter of the brachial artery before the introduction of a flow stimulus in the artery. The post occlusion arterial diameter is the diameter observed within minutes of reperfusion following the release of an inflated cuff.

For crossover trials, the net response in FMD was calculated by subtracting the mean FMD value at the end of the control period from the mean FMD value at the end of the treatment period. For parallel studies, mean changes in the control group were subtracted from mean changes in the intervention group. Mean changes were defined as the difference between measurements before (start-of-the-study values) and after the study (end-of-the-study values). For trials in which different doses of vitamin supplements were supplied or that performed FMD measurements more than one time during the study, multiple study arms were considered.

As described in [12], summary estimates of weighted mean differences (WMDs) in FMD and 95% confidence intervals (CIs) were calculated using fixed-effect meta-analyses and visualized using forest plots. The inverse of the variance (1/SE^2^) (SE = within-study variance) was used as weight factor. Heterogeneity was evaluated using the Cochran’s Q test (*p* < 0.1 indicates statistical significant heterogeneity) and quantified using the I^2^ statistic [13,14,15], *i.e.*, the percentage of variability in effect estimate that is due to heterogeneity rather than sampling error. An I^2^ value above 50% indicates relevant heterogeneity between studies [16]. In case of heterogeneity, random-effect meta-analyses were used as described by DerSimonian and Laird [14].

As it was evident that the type of vitamin supplement was an important source of heterogeneity, first a vitamin E and a vitamin D group were defined. Subgroup analyses were performed within each vitamin group to identify sources of heterogeneity between studies by comparing the summary results of the study arms grouped by subject characteristics (*i.e*., mean age, gender, baseline BMI, baseline FMD level and health status), treatment characteristics (*i.e*., type of vitamin supplement, total supplement dose and duration of follow-up) and technical aspects of the FMD measurement (position of cuff and time of occlusion). Median values of continuous variables were used as cutoff values to create the binary variables. Univariate meta-regression analysis was performed to investigate the effect of the dose of the vitamin supplement and other characteristics on the change in FMD. For all statistical analyses, two-sided tests were used. Statistical significance was set at *p* < 0.05. Publication bias was finally evaluated visually by inspecting the symmetry of funnel plots. The degree of funnel plot asymmetry was assessed with the Egger’s weighted regression test. Absence of publication bias is reflected in an intercept close to 0 with a corresponding *p* ≥ 0.05 [17].

## 3. Results

### 3.1. Search Results and Study Selection

**Figure 1:**
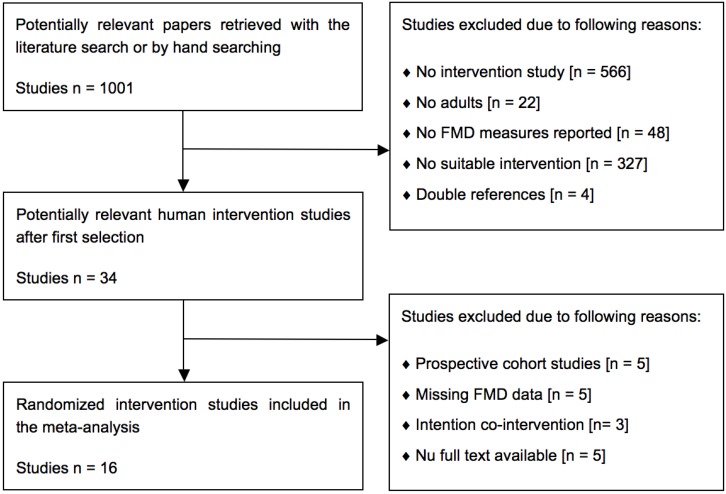
Flow diagram showing the study selection procedure of human intervention studies for the meta-analysis of fat-soluble vitamin supplements and endothelial function as measured by flow-mediated vasodilation (FMD), with the specification of reasons.

A total of 1001 potentially relevant papers were retrieved with the systematic search. Based on the predefined selection criteria, 967 papers were excluded for different reasons (Figure 1). The full texts of the remaining 34 articles were reviewed and eighteen papers were excluded for the following reasons: prospective cohort studies [18,19,20,21,22], missing data on FMD [23,24,25,26,27], no appropriate measures of variability reported [18], intentional co-intervention [28,29,30], or no full text available (five conference abstracts). A total of sixteen RCTs in adult volunteers with parallel [31,32,33,34,35,36,37,38,39,40,41,42,43,44,45] or crossover designs [46] met all the inclusion criteria and were finally included (Table 1). In seven studies, effects of vitamin E [31,32,35,37,38,39,46] were examined and in nine studies, those of vitamin D [33,34,36,40,41,42,43,44,45]. No intervention studies were found that examined the effects of carotenoid or vitamin K supplements.

### 3.2. Study Characteristics

In six parallel studies [31,32,35,37,38,39] and in one crossover [46] trial, effects of vitamin E were examined. These studies provided eight relevant study arms and included 303 subjects. The number of subjects per study ranged from 20 to 70, the mean age of the participants from 23.0 to 59.8 years, and the mean BMI from 23.1 to 29.2 kg/m^2^. The mean baseline FMD was 4.40% (range: 1.83% to 6.20%). Two studies included only men [31,37], whereas the proportion of men in the remaining studies ranged from 40.0% to 60.0%. In two studies, healthy elderly [46] or healthy smoking adults [37] were included, in three studies patients with diabetes [32,38,39], whereas in two studies subjects had hypercholesterolemia [31] or atypical chest pain [35]. Study duration varied between four weeks and twelve months, and the daily dose of vitamin E from 300 to 1800 IU (mean: 1090 IU/day; Table 1).

For vitamin D, nine RCTs with parallel designs [33,34,36,40,41,42,43,44,45] were identified including a total of fifteen study arms. In these studies, 658 subjects participated, 345 in the treatment groups and 313 in the control groups. The mean age of the subjects was 59.8 years (range: 29.0 to 76.9 years), BMI was 28.4 kg/m^2^ (range: 24.9 to 31.7 kg/m^2^), and half of the study population was men. In three studies, healthy women [33,41] or African American adults [34] were included; the other studies included subjects with type II diabetes [40,42,45], isolated systolic hypertension [44], HIV infection [36], or a history of stroke [43]. In two studies [40,43], a single dose of 100,000 IU of vitamin D2 was administered. The dose of the vitamin D3 supplement in the other studies ranged from 2000 to 5000 IU/day [33,34,36,45], or subjects received a total of 100,000 to 200,000 IU once or every three months [41,42,44]. Study duration ranged from four weeks to twelve months (Table 1).

**Table 1:** Overview of randomized placebo-controlled trials included in the meta-analysis.

First Author and Year	Subject Characteristics						Treatment Characteristics			FMD Measurement Characteristics		
	Design ^1^	Number	Male (%)	Age (Y)	BMI (kg/m^2^)	Health Status	Treatment ^2^	Dose ^3^	Duration	Cuff	Occlusion	Baseline (%)		
Randomized controlled trials (RCTs) with vitamin E supplements as experimental variable ^4^																		
Borovničar, 2000 (31)	P	22/22	100/100	45.0/45.0	-/-	High cholesterol	Toco-acetate	889 IU/day	6 months	Distal	4.0 min	6.20/6.50
Economides, 2005 (32)	P	32/31	53.9/53.9	53.0/53.0	29.2/29.2	Type I/II diabetes	All-racemic α-Toc	1800 IU/day	12 months	-	-	5.80/5.80
Economides, 2005 (32)	P	34/32	53.9/53.9	53.0/53.0	29.2/29.2	Type I/II diabetes	All-racemic α-Toc	1800 IU/day	6 months	-	-	5.70/5.70
Kugiyama, 1999 (35)	P	35/35	40.0/45.7	59.8/60.1	23.1/23.1	Chest pain	α-Toco-acetate	300 IU/day	4 weeks	Distal	5.0 min	5.04/5.02
Neunteufl, 2000 (37)	P	11/11	100/100	28.0/27.0	-/-	Smokers	All-racemic α-Toc	600 IU/day	4 weeks	Proximal	4.5 min	5.30/6.40
Paolisso, 2000 (38)	P	20/20	45.0/60.0	58.3/56.7	27.6/27.4	Type II diabetes	Vitamin E	1333 IU/day	8 weeks	Proximal	5.5 min	1.83/1.71
Simons, 1999 (46)	CO	20/-	65.0/-	57.0/-	27.5/-	Healthy	RRR D-α-Toco	1000 IU/day	10 weeks	Distal	4.5 min	2.70/-
Skyrme-Jones, 2000 (39)	P	20/21	47.6/45.0	23.0/28.0	24.3/25.5	Type I diabetes	All-racemic α-Toc	1000 IU/day	3 months	-	5.0 min	2.60/2.40
Randomized controlled trials (RCTs) with vitamin D supplements as experimental variable ^5^																		
Gepner, 2012 (33)	P	55/55	00.0/00.0	64.1/63.6	27.1/25.3	Post-menopausal	Vitamin D3	2500 IU/day	4 months	Distal	5.0 min	5.05/4.57
Harris, 2011 (34)	P	22/23	41.0/52.0	29.0/31.0	30.4/29.1	African American	Vitamin D3	2000 IU/day	16 weeks	Distal	5.0 min	7.23/6.55
Longenecker, 2012 (36)	P	30/15	83.3/66.7	47.0/10.0	28.0/27.0	HIV-infected	Vitamin D3	4000 IU/day	12 weeks	Distal	5.0 min	2.87/2.46
Sugden, 2008 (40)	P	17/17	58.8/47.1	64.9/63.5	31.7/31.7	Type II diabetes	Vitamin D2	100K IU (0)	8 weeks	Distal	5.0 min	6.38/7.28
Witham, 2010 (42)	P	19/21	84.2/54.5	65.3/66.7	31.1/33.3	Type II diabetes	Vitamin D3	100K IU (0)	16 weeks	Distal	-	5.10/5.40
Witham, 2010 (42)	P	18/21	65.0/54.5	63.3/66.7	29.7/33.3	Type II diabetes	Vitamin D3	200K IU (0)	16 weeks	Distal	-	6.40/5.40
Witham, 2010 (42)	P	19/22	84.2/54.5	65.3/66.7	31.1/33.3	Type II diabetes	Vitamin D3	100K IU (0)	8 weeks	Distal	-	5.10/5.40
Witham, 2010 (42)	P	17/22	65.0/54.5	63.3/66.7	29.7/33.3	Type II diabetes	Vitamin D3	200K IU (0)	8 weeks	Distal	-	6.40/5.40
Witham, 2012 (43)	P	28/27	60.0/85.7	66.2/67.7	27.3/26.1	History stroke	Vitamin D2	100K IU (0)	16 weeks	Distal	5.0 min	6.90/5.60
Witham, 2012 (43)	P	29/27	60.0/85.7	66.2/67.7	27.3/26.1	History stroke	Vitamin D2	100K IU (0)	8 weeks	Distal	5.0 min	6.90/5.60
Witham, 2013 (44)	P	80/79	50.0/53.2	76.9/76.7	28.5/27.9	High systolic BP	Vitamin D3	100K IU (3)	12 months	Distal	5.0 min	5.10/5.10
Witham, 2013 (44)	P	80/79	50.0/53.2	76.9/76.7	28.5/27.9	High systolic BP	Vitamin D3	100K IU (0)	3 months	Distal	5.0 min	5.10/5.10
Witham, 2013 (41)	P	25/25	00.0/00.0	41.7/39.4	24.9/28.7	South Asian	Vitamin D3	100K IU (0)	8 weeks	Distal	5.0 min	8.20/8.70
Witham, 2013 (41)	P	25/25	00.0/00.0	41.7/39.4	24.9/28.7	South Asian	Vitamin D3	100K IU (0)	4 weeks	Distal	5.0 min	8.20/8.70
Yiu, 2013 (45)	P	50/50	54.0/46.0	65.8/64.9	25.8/25.1	Type II diabetes	Vitamin D3	5000 IU/day	12 weeks	Distal	5.0 min	3.39/3.40

^1^ Study design: randomized placebo-controlled parallel (P) or crossover (CO) trials; ^2^ Study treatments as described by the authors. Toc: tocopherol, Vitamin D2: oral ergocalciferol, Vitamin D3: oral cholecalciferol; ^3^ Doses in mg were transformed to international units (IU) according to vitamin E guidelines. Supplement received once (0) or every three months (3); ^4^ *n* = 7 intervention studies (experimental group/control group) with eight relevant study arms; ^5^ *n* = 9 intervention studies (experimental group/control group) with 15 relevant study arms.

### 3.3. Effect of Vitamin Supplementation on FMD

**Figure 2:**
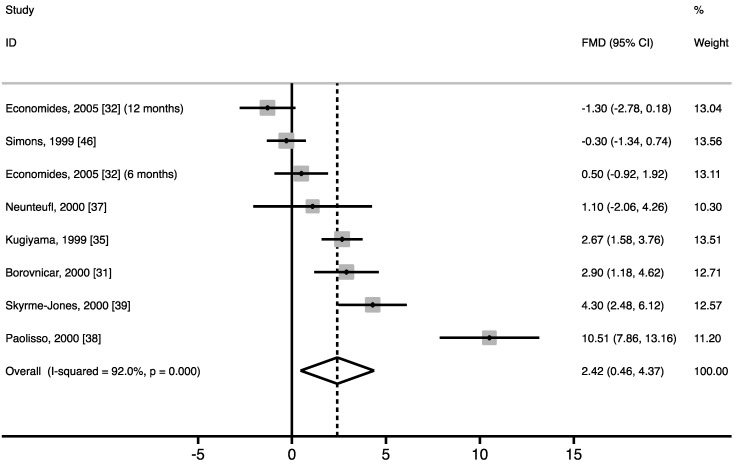
Forest plot of random controlled trials (RCTs) that investigated the effect of vitamin E supplements on flow-mediated vasodilation (FMD). The solid squares represent the weight of individual studies and the diamond represents the weighted mean difference (WMD) in FMD (calculated using random-effect meta-analyses). In all studies combined, vitamin E increased FMD *vs*. control by 2.42% (95% CI: 0.46% to 4.37%; *p* = 0.015). After excluding the study by Paolisso and colleagues [38], the overall WMD nearly reached statistical significance (1.38%; 95% CI: −0.12% to 2.87%; *p* = 0.070).

Vitamin E supplementation increased fasting FMD *vs*. control by 2.42% (95% CI: 0.46% to 4.37%; *p* = 0.015) (Figure 2), corresponding to a relative increase of approximately 50% compared with baseline FMD values. However, significant heterogeneity was found (I^2^ = 92.0%, *p* < 0.001). Results of the meta-regression analysis showed no linear dose-response relationship between the dose of the vitamin E supplement and the change in FMD (*p* = 0.774; Figure S1). Analyses were repeated after excluding the study of Paolisso and colleagues [38], which had the most pronounced effect (10.5%). The overall WMD in FMD decreased and nearly reached statistical significance (1.38%; 95% CI: −0.12% to 2.87%; *p* = 0.070). No linear dose-response relationship was found between the dose of the vitamin E supplement and the change in FMD (*p* = 0.161). Even though heterogeneity decreased, it remained significant (I^2^ = 85.8%, *p* < 0.001).

**Figure 3:**
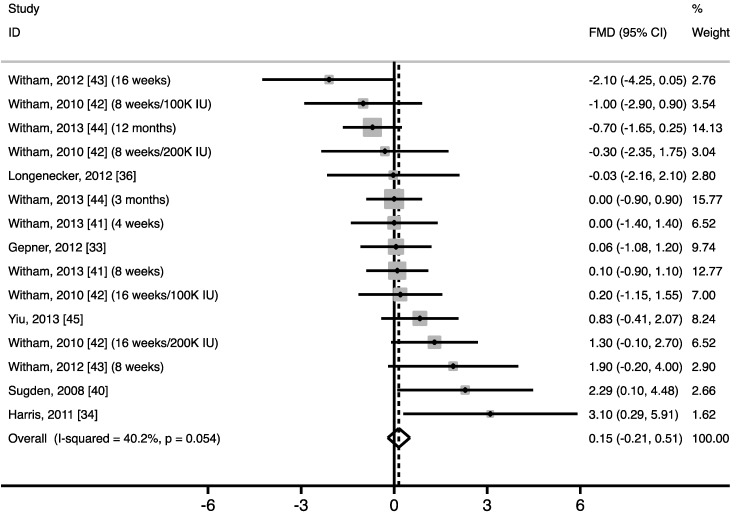
Forest plot of random controlled trials (RCTs) that investigated the effect of vitamin D supplements on flow-mediated vasodilation (FMD). The solid squares represent the weight of individual studies and the diamond represents the weighted mean difference (WMD) in FMD (calculated using fixed-effect meta-analyses). In all studies combined, vitamin D did not increase FMD (WMD: 0.15%; 95% CI: −0.21% to 0.51%; *p* = 0.41).

Vitamin D supplementation did not significantly improve fasting FMD of the brachial artery *vs*. control (0.15%; 95% CI: −0.21% to 0.51%; *p* = 0.41) (Figure 3). Between-study heterogeneity nearly reached statistical significance (I^2^ = 40.2%, *p* = 0.054). After using only results of the last measurement for trials that performed FMD measurements more than one time during the study [41,42,43,44], similar results were found for the overall WMD (0.33%; 95% CI: −0.34% to 1.00%; *p* = 0.34). Heterogeneity, however, became statistically significant (I^2^ = 52.5%, *p* = 0.026).

### 3.4. Subgroup Analyses

Subgroup analyses were performed within each vitamin group to evaluate if subject characteristics, treatment characteristics or technical aspects of the FMD measurement were related to the effects observed (Table 2). None of the predefined variables resulted in significantly different effects of fat-soluble vitamin supplement intake on FMD between the subgroups. Further, the impact of baseline plasma 25-hydroxyvitamin D (25(OH)-D) concentrations and vitamin D-induced changes in 25(OH)-D was investigated, but no association with the change in fasting FMD was found (data not shown).

### 3.5. Publication Bias

The funnel plot for the effect of vitamin E supplementation did not reveal possible presence of publication bias (Figure S2) and the Egger’s weighted regression test also showed no funnel plot asymmetry (*p* = 0.20). Similar results were found after visual evaluation of the funnel plot for the effect of vitamin D supplementation and the Egger’s test (*p* = 0.24) did not reach statistical significance, indicating absence of publication bias (Figure S3).

**Table 2:** Subgroup analyses for the effect of fat-soluble vitamin E and vitamin D supplementation on fasting flow-mediated vasodilation (FMD) in adults.

Study Characteristic	Mean	Stratification Variable	No of Study Arms	WMD (%) ^1^	95% CI (%)	*p*-value Difference
Study arms with vitamin E supplements as experimental variable ^2^						
Mean age (years) ^3^	47.1	≤53.0	5	1.48	−0.59 to 3.55	0.372
		>53.0	3	4.08	−0.25 to 8.41	
Gender (% male) ^3^	63.2	≤53.9	5	3.17	0.25 to 6.10	0.500
		>53.9	3	1.17	−1.14 to 3.49	
Baseline BMI (kg/m^2^) ^3^	26.8	≤27.5	3	2.15	−0.43 to 4.72	0.828
		>27.5	3	3.11	−2.39 to 8.60	
Baseline FMD (%) ^3^	4.40	≤5.17	4	4.10	0.71 to 7.50	0.219
		>5.17	4	0.74	−1.16 to 2.63	
Health status	-	Healthy	2	−0.16	−1.15 to 0.82	0.390
		Diseased	6	3.10	0.67 to 5.54	
Dose (100 IU/day) ^3^	10.9	≤10.0	5	2.14	0.35 to 3.93	0.764
		>10.0	3	3.11	−2.39 to 8.60	
Study duration (weeks) ^3^	16.8	≤11.0	4	3.39	−0.19 to 6.97	0.523
		>11.0	4	1.56	−0.83 to 3.94	
Position cuff	-	Distal	3	1.40	0.71 to 2.09	0.341
		Proximal	2	6.63	4.60 to 8.66	
Occlusion duration (min) ^4^	4.67	≤4.50	3	1.17	−1.14 to 3.49	0.158
		>4.50	3	5.65	1.77 to 9.54	
Study arms with vitamin D supplements as experimental variable ^4^						
Mean age (years) ^3^	59.8< 44.9	≤64.9	8	0.45	−0.09 to 0.98	0.263
		>64.9	7	−0.10	−0.59 to 0.38	
Gender (% male) ^3^	50.4	≤58.8	8	0.14	−0.28 to 0.56	0.805
		>58.8	7	0.18	−0.49 to 0.85	
Baseline BMI (kg/m^2^) ^3^	28.4	≤28.5	9	−0.02	−0.43 to 0.39	0.226
		>28.5	6	0.68	−0.05 to 1.40	
Baseline FMD (%) ^3^	5.89	≤6.38	8	0.02	−0.43 to 0.47	0.550
		>6.38	7	0.38	−0.22 to 0.97	
Health status	-	Healthy	4	0.23	−0.42 to 0.87	0.820
		Diseased	11	0.12	−0.31 to 0.55	
Type of vitamin ^5^	-	Vitamin D2	3	0.70	−0.54 to 1.94	0.521
		Vitamin D3	12	0.10	−0.27 to 0.47	
Study duration (weeks) ^3^	13.9	≤12.0	9	0.26	−0.21 to 0.73	0.675
		>12.0	6	−0.01	−0.56 to 0.55	
Position cuff	-	Distal	15	0.15	−0.21 to 0.51	-
		Proximal	0	-	-	
Occlusion duration (min) ^4^	5.00	≤5.00	11	0.12	−0.28 to 0.52	-
		>5.00	0	-	-	

^1^ WMD: Weighted mean difference in flow-mediated vasodilation; ^2^ *n* = 7 Intervention studies with eight relevant study arms; ^3^ Study arms were divided into subgroups based on their medians; ^4^ *n* = 9 intervention studies with 15 relevant study arms; ^5^ Vitamin D2: oral ergocalciferol, Vitamin D3: oral cholecalciferol.

## 4. Discussion

In this meta-analysis, data from seven RCTs studying the effects of vitamin E supplements and from nine RCTs examining the effects of vitamin D supplements on fasting FMD in adults were pooled. When all studies were included, we found that vitamin E supplementation increased FMD of the brachial artery by 2.42%, while no effect of vitamin D supplementation was found. However, significant heterogeneity was found among the vitamin E trials.

The putative positive effects of vitamin E on FMD may be due to increased scavenging of oxygen free radicals by vitamin E or via improved plasma antioxidant defenses [38]. This may lower the quenching effect of free radicals on nitric oxide (NO), thereby improving NO bioavailability and endothelial function. Several studies, but not all [47,48], have further suggested that vitamin E supplementation increases insulin sensitivity [49,50], which may also improve endothelial function [39]. However, based on our results, the use of vitamin E supplements to improve FMD should still be questioned. First, no linear dose-response relationship was evident, which would have been strong evidence for a causal relationship. Also, the level of vitamin E tested in RCTs included in our meta-analysis ranged from 300 to 1800 IU/day, which is far above recommended intakes of 30 IU/day [51]. In fact, concern has been raised about potential adverse effects. One meta-analysis indeed concluded that high-dose vitamin E supplementation may increase all-cause mortality in trials supplying daily doses over 400 IU, that involved adults with chronic diseases [52]. In another meta-analysis, vitamin E given singly or in combination with other antioxidant supplements was also found to increase mortality in randomized primary and secondary prevention trials when trials with low methodological quality were excluded [53]. Further, the increment in FMD was not longer statistically significant when the study with the most pronounced effect was excluded [38]. The most pronounced effect (10.5%) substantially differed from the overall WMD in FMD of 2.42%. However, in that study, subjects had the lowest mean baseline FMD (2.00%), suggesting that a very specific population was studied. Participants were type II diabetics, but it is not likely that this explains the large effects. Two other studies were also carried out in diabetic patients with both lower (1000 IU) [39] and higher daily doses (1800 IU) of vitamin E [32], but effects were never as large as observed by Paolisso and colleagues [38]. Considering these uncertainties, the present results should therefore not be interpreted as conclusive evidence to support the use of vitamin E supplements to improve FMD.

Prospective cohort studies have reported associations between vitamin D deficiency [54] or low plasma 25(OH)-D [55] and incident CVD. Although *in vitro* and animal studies have suggested a role of vitamin D supplements on endothelial function [56], we found no effects of vitamin D supplements on fasting FMD in adults. Another possible explanation for the inverse relation between vitamin D and CVD may be through effects on blood pressure. However, effects of vitamin D on blood pressure are also inconclusive [8]. In addition, calcium intakes may modify possible effects of vitamin D status on associated health benefits [57], and concomitant calcium intake may thus be required to observe positive effects of vitamin D supplementation on fasting vascular endothelial function.

Finally, a number of observational studies have reported an inverse association between carotenoid intake and CVD risk [58]. However, intervention trials using β-carotene supplements have not supported the hypothesis that β-carotene reduces CVD [53,58]. Unfortunately, we found no studies that examined the effects of carotenoid supplements on FMD. Further, an inverse association between vitamin K2 intake and coronary heart disease was found [59] and an increased dietary intake of vitamin K was also associated with a reduced risk of cardiovascular mortality [60]. However, results from intervention trials are lacking and none of the included trials investigated the effect of vitamin K supplementation on brachial reactivity.

Significant heterogeneity was found between vitamin E trials and results of previous studies were indeed inconsistent. It has been speculated that various factors, including the mean age of participants [32,61], vitamin supplement dose [23,32,61] or duration of the intervention [23,32,61], may explain these inconsistent results. However, none of these or other predefined variables accounted for the variable effect of vitamin E supplementation interventions on FMD. Montero and colleagues found that effects of antioxidant vitamin supplementation on endothelial function did depend on the BMI of subjects [6]. Positive effects were only found in non-obese subjects, which were explained by an insufficient capacity of oral vitamin E intakes to overcome increased levels of oxidative stress [62]. However, only studies involving subjects with type II diabetes were included and effects of antioxidant vitamin E and vitamin C could not be separated in the intervention studies selected. Further, measurements of vascular endothelial function from different vascular regions were pooled and effects on FMD as such were not quantified. After vitamin D supplementation, there were also no predefined characteristics that were significantly related to the effects observed on fasting FMD. In an additional analysis, no association with baseline plasma 25(OH)-D concentrations or vitamin D-induced changes in 25(OH)-D was found. However, heterogeneity among vitamin D studies did not reach statistical significance.

A possible limitation of our meta-analysis is the variability in experimental designs that may have contributed to the heterogeneity observed among the included studies. Further, comparability of FMD measurements between centers is low [63] and may be another source of heterogeneity. Even though technical aspects of the FMD measurement were not significantly related to the effects observed, better standardization of the FMD technique is required to reduce variability between trials included in the meta-analysis.

## 5. Conclusions

In conclusion, a causal relationship between high-dose vitamin E supplementation and improved fasting FMD cannot be ascertained from the present meta-analysis, while no effects of vitamin D supplementation were found. Therefore, the current meta-analysis does not provide unambiguous evidence to support the use of fat-soluble vitamin supplements to improve vascular endothelial function in adults and more research is required.

## Supplementary Files

Supplementary File 1

## References

[1] Lichtenstein A.H. (2009). Nutrient supplements and cardiovascular disease: A heartbreaking story. J. Lipid Res..

[2] Widlansky M.E., Gokce N., Keaney J.F., Vita J.A. (2003). The clinical implications of endothelial dysfunction. J. Am. Coll. Cardiol..

[3] Schachinger V., Britten M.B., Zeiher A.M. (2000). Prognostic impact of coronary vasodilator dysfunction on adverse long-term outcome of coronary heart disease. Circulation.

[4] Cohn J.N., Quyyumi A.A., Hollenberg N.K., Jamerson K.A. (2004). Surrogate markers for cardiovascular disease: Functional markers. Circulation.

[5] Ellins E.A., Halcox J.P. (2011). Where are we heading with noninvasive clinical vascular physiology? Why and how should we assess endothelial function?. Cardiol. Res. Pract..

[6] Montero D., Walther G., Stehouwer C.D., Houben A.J., Beckman J.A., Vinet A. (2014). Effect of antioxidant vitamin supplementation on endothelial function in type 2 diabetes mellitus: A systematic review and meta-analysis of randomized controlled trials. Obes Rev..

[7] Ashor A.W., Lara J., Mathers J.C., Siervo M. (2014). Effect of vitamin C on endothelial function in health and disease: A systematic review and meta-analysis of randomised controlled trials. Atherosclerosis.

[8] Min B. (2013). Effects of vitamin D on blood pressure and endothelial function. Korean J. Physiol. Pharmacol..

[9] Liu Z.M., Woo J., Wu S.H., Ho S.C. (2013). The role of vitamin D in blood pressure, endothelial and renal function in postmenopausal women. Nutrients.

[10] Liberati A., Altman D.G., Tetzlaff J., Mulrow C., Gotzsche P.C., Ioannidis J.P., Clarke M., Devereaux P.J., Kleijnen J., Moher D. (2009). The PRISMA statement for reporting systematic reviews and meta-analyses of studies that evaluate healthcare interventions: Explanation and elaboration. BMJ.

[11] Arab L., Barr S.I., Becking G.C. (2000). A Report of the Panel on Dietary Antioxidants and Related Compounds. Dietary Reference Intakes for Vitamin C, Vitamin E, Selenium, and Carotenoids.

[12] Joris P.J., Zeegers M.P., Mensink R.P. (2014). Weight loss improves fasting flow-mediated vasodilation in adults: A meta-analysis of intervention studies. Atherosclerosis.

[13] Cochran W.G. (1954). The combination of estimates from different experiments. Biometrics.

[14] DerSimonian R., Laird N. (1986). Meta-analysis in clinical trials. Control. Clin. Trials.

[15] Higgins J.P., Thompson S.G. (2002). Quantifying heterogeneity in a meta-analysis. Stat. Med..

[16] Higgins J.P., Thompson S.G., Deeks J.J., Altman D.G. (2003). Measuring inconsistency in meta-analyses. BMJ.

[17] Egger M., Davey S.G., Schneider M., Minder C. (1997). Bias in meta-analysis detected by a simple, graphical test. BMJ.

[18] Can M., Gunes M., Haliloglu O.A., Haklar G., Inanc N., Yavuz D.G., Direskeneli H. (2012). Effect of vitamin D deficiency and replacement on endothelial functions in Behçet's disease. Clin. Exp. Rheumatol..

[19] Chitalia N., Ismail T., Tooth L., Boa F., Hampson G., Goldsmith D., Kaski J.C., Banerjee D. (2014). Impact of vitamin D supplementation on arterial vasomotion, stiffness and endothelial biomarkers in chronic kidney disease patients. PLoS One.

[20] Koh K.K., Blum A., Hathaway L., Mincemoyer R., Csako G., Waclawiw M.A., Panza J.A., Cannon R.O. (1999). Vascular effects of estrogen and vitamin E therapies in postmenopausal women. Circulation.

[21] Stein J.H., Carlsson C.M., Papcke-Benson K., Aeschlimann S.E., Bodemer A., Carnes M., McBride P.E. (2001). The effects of lipid-lowering and antioxidant vitamin therapies on flow-mediated vasodilation of the brachial artery in older adults with hypercholesterolemia. J. Am. Coll. Cardiol..

[22] Tarcin O., Yavuz D.G., Ozben B., Telli A., Ogunc A.V., Yuksel M., Toprak A., Yazici D., Sancak S., Deyneli O. (2009). Effect of vitamin D deficiency and replacement on endothelial function in asymptomatic subjects. J. Clin. Endocrinol. Metab..

[23] Gazis A., White D.J., Page S.R., Cockcroft J.R. (1999). Effect of oral vitamin E (alpha-tocopherol) supplementation on vascular endothelial function in type 2 diabetes mellitus. Diabet Med..

[24] Green D., OʼDriscoll G., Rankin J.M., Maiorana A.J., Taylor R.R. (1998). Beneficial effect of vitamin E administration on nitric oxide function in subjects with hypercholesterolaemia. Clin. Sci. (Lond).

[25] Pinkney J.H., Downs L., Hopton M., Mackness M.I., Bolton C.H. (1999). Endothelial dysfunction in type 1 diabetes mellitus: Relationship with LDL oxidation and the effects of vitamin E. Diabet Med..

[26] Shab-Bidar S., Neyestani T.R., Djazayery A., Eshraghian M.R., Houshiarrad A., Gharavi A., Kalayi A., Shariatzadeh N., Zahedirad M., Khalaji N. (2011). Regular consumption of vitamin D-fortified yogurt drink (Doogh) improved endothelial biomarkers in subjects with type 2 diabetes: A randomized double-blind clinical trial. BMC Med..

[27] Witham M.D., Dove F.J., Khan F., Lang C.C., Belch J.J., Struthers A.D. (2013). Effects of vitamin D supplementation on markers of vascular function after myocardial infarction-a randomised controlled trial. Int. J. Cardiol..

[28] Mah E., Pei R., Guo Y., Ballard K.D., Barker T., Rogers V.E., Parker B.A., Taylor A.W., Traber M.G., Volek J.S. (2013). γ-Tocopherol-rich supplementation additively improves vascular endothelial function during smoking cessation. Free Radic. Biol. Med..

[29] Motoyama T., Kawano H., Kugiyama K., Hirashima O., Ohgushi M., Tsunoda R., Moriyama Y., Miyao Y., Yoshimura M., Ogawa H. (1998). Vitamin E administration improves impairment of endothelium-dependent vasodilation in patients with coronary spastic angina. J. Am. Coll. Cardiol..

[30] Neunteufl T., Kostner K., Katzenschlager R., Zehetgruber M., Maurer G., Weidinger F. (1998). Additional benefit of vitamin E supplementation to simvastatin therapy on vasoreactivity of the brachial artery of hypercholesterolemic men. J. Am. Coll. Cardiol..

[31] Borovnicar A., Keber I., Stavljenic Rukavina A., Yaletel Kragelj L. (2000). Improvement of early functional atherosclerotic changes in males with hypercholesterolemia after vitamin E supplementation. Pflugers Arch..

[32] Economides P.A., Khaodhiar L., Caselli A., Caballero A.E., Keenan H., Bursell S.E., King G.L., Johnstone M.T., Horton E.S., Veves A. (2005). The effect of vitamin E on endothelial function of micro- and macrocirculation and left ventricular function in type 1 and type 2 diabetic patients. Diabetes.

[33] Gepner A.D., Ramamurthy R., Krueger D.C., Korcarz C.E., Binkley N., Stein J.H. (2012). A prospective randomized controlled trial of the effects of vitamin D supplementation on cardiovascular disease risk. PLoS One.

[34] Harris R.A., Pedersen-White J., Guo D.H., Stallmann-Jorgensen I.S., Keeton D., Huang Y., Shah Y., Zhu H., Dong Y. (2011). Vitamin D3 supplementation for 16 weeks improves flow-mediated dilation in overweight African-American adults. Am. J. Hypertens.

[35] Kugiyama K., Motoyama T., Doi H., Kawano H., Hirai N., Soejima H., Miyao Y., Takazoe K., Moriyama Y., Mizuno Y. (1999). Improvement of endothelial vasomotor dysfunction by treatment with alpha-tocopherol in patients with high remnant lipoproteins levels. J. Am. Coll. Cardiol..

[36] Longenecker C.T., Hileman C.O., Carman T.L., Ross A.C., Seydafkan S., Brown T.T., Labbato D.E., Storer N., Tangpricha V., McComsey G.A. (2012). Vitamin D supplementation and endothelial function in vitamin D deficient HIV-infected patients: A randomized placebo-controlled trial. Antivir. Ther..

[37] Neunteufl T., Priglinger U., Heher S., Zehetgruber M., Soregi G., Lehr S., Huber K., Maurer G., Weidinger F., Kostner K. (2000). Effects of vitamin E on chronic and acute endothelial dysfunction in smokers. J. Am. Coll. Cardiol..

[38] Paolisso G., Tagliamonte M.R., Barbieri M., Zito G.A., Gambardella A., Varricchio G., Ragno E., Varricchio M. (2000). Chronic vitamin E administration improves brachial reactivity and increases intracellular magnesium concentration in type II diabetic patients. J. Clin. Endocrinol. Metab..

[39] Skyrme-Jones R.A., O’Brien R.C., Berry K.L., Meredith I.T. (2000). Vitamin E supplementation improves endothelial function in type I diabetes mellitus: A randomized, placebo-controlled study. J. Am. Coll. Cardiol..

[40] Sugden J.A., Davies J.I., Witham M.D., Morris A.D., Struthers A.D. (2008). Vitamin D improves endothelial function in patients with type 2 diabetes mellitus and low vitamin D levels. Diabet Med..

[41] Witham M.D., Adams F., Kabir G., Kennedy G., Belch J.J., Khan F. (2013). Effect of short-term vitamin D supplementation on markers of vascular health in South Asian women living in the UK—A randomised controlled trial. Atherosclerosis.

[42] Witham M.D., Dove F.J., Dryburgh M., Sugden J.A., Morris A.D., Struthers A.D. (2010). The effect of different doses of vitamin D(3) on markers of vascular health in patients with type 2 diabetes: A randomised controlled trial. Diabetologia.

[43] Witham M.D., Dove F.J., Sugden J.A., Doney A.S., Struthers A.D. (2012). The effect of vitamin D replacement on markers of vascular health in stroke patients—A randomised controlled trial. Nutr. Metab. Cardiovas. Dis..

[44] Witham M.D., Price R.J., Struthers A.D., Donnan P.T., Messow C.M., Ford I., McMurdo M.E. (2013). Cholecalciferol treatment to reduce blood pressure in older patients with isolated systolic hypertension: The VitDISH randomized controlled trial. JAMA Intern. Med..

[45] Yiu Y.F., Yiu K.H., Siu C.W., Chan Y.H., Li S.W., Wong L.Y., Lee S.W., Tam S., Wong E.W., Lau C.P. (2013). Randomized controlled trial of vitamin D supplement on endothelial function in patients with type 2 diabetes. Atherosclerosis.

[46] Simons L.A., von Konigsmark M., Simons J., Stocker R., Celermajer D.S. (1999). Vitamin E ingestion does not improve arterial endothelial dysfunction in older adults. Atherosclerosis.

[47] Shab-Bidar S., Mazloum Z., Mousavi-Shirazifard Z. (2013). Daily vitamin E supplementation does not improve metabolic and glycemic control in type 2 diabetic patients: A double blinded randomized controlled trial. J. Diabetes.

[48] de Oliveira A.M., Rondo P.H., Luzia L.A., DʼAbronzo F.H., Illison V.K. (2011). The effects of lipoic acid and alpha-tocopherol supplementation on the lipid profile and insulin sensitivity of patients with type 2 diabetes mellitus: A randomized, double-blind, placebo-controlled trial. Diabetes Res. Clin. Pract..

[49] Paolisso G., DʼAmore A., Giugliano D., Ceriello A., Varricchio M., DʼOnofrio F. (1993). Pharmacologic doses of vitamin E improve insulin action in healthy subjects and non-insulin-dependent diabetic patients. Am. J. Clin. Nutr..

[50] Paolisso G., di Maro G., Galzerano D., Cacciapuoti F., Varricchio G., Varricchio M., D’Onofrio F. (1994). Pharmacological doses of vitamin E and insulin action in elderly subjects. Am. J. Clin. Nutr..

[51] Monsen E.R. (2000). Dietary reference intakes for the antioxidant nutrients: Vitamin C, vitamin E, selenium, and carotenoids. J. Am. Diet. Assoc..

[52] Miller E.R., Pastor-Barriuso R., Dalal D., Riemersma R.A., Appel L.J., Guallar E. (2005). Meta-analysis: High-dosage vitamin E supplementation may increase all-cause mortality. Ann. Intern. Med..

[53] Bjelakovic G., Nikolova D., Gluud L.L., Simonetti R.G., Gluud C. (2007). Mortality in randomized trials of antioxidant supplements for primary and secondary prevention: Systematic review and meta-analysis. JAMA.

[54] Wang T.J., Pencina M.J., Booth S.L., Jacques P.F., Ingelsson E., Lanier K., Benjamin E.J., DʼAgostino R.B., Wolf M., Vasan R.S. (2008). Vitamin D deficiency and risk of cardiovascular disease. Circulation.

[55] Dobnig H., Pilz S., Scharnagl H., Renner W., Seelhorst U., Wellnitz B., Kinkeldei J., Boehm B.O., Weihrauch G., Maerz W. (2008). Independent association of low serum 25-hydroxyvitamin D and 1,25-dihydroxyvitamin D levels with all-cause and cardiovascular mortality. Arch. Intern. Med..

[56] Dalan R., Liew H., Tan A.W., Chew D.E., Leow M.K. (2014). Vitamin D and the endothelium: Basic, translational and clinical research updates. IJC Metab. Endocr..

[57] Heaney R.P. (2008). Vitamin D and calcium interactions: Functional outcomes. Am. J. Clin. Nutr..

[58] Voutilainen S., Nurmi T., Mursu J., Rissanen T.H. (2006). Carotenoids and cardiovascular health. Am. J. Clin. Nutr..

[59] Geleijnse J.M., Vermeer C., Grobbee D.E., Schurgers L.J., Knapen M.H., van der Meer I.M., Hofman A., Witteman J.C. (2004). Dietary intake of menaquinone is associated with a reduced risk of coronary heart disease: The Rotterdam Study. J. Nutr..

[60] Juanola-Falgarona M., Salas-Salvado J., Martinez-Gonzalez M.A., Corella D., Estruch R., Ros E., Fito M., Aros F., Gomez-Gracia E., Fiol M. (2014). Dietary intake of vitamin K is inversely associated with mortality risk. J. Nutr..

[61] Lu Q., Bjorkhem I., Wretlind B., Diczfalusy U., Henriksson P., Freyschuss A. (2005). Effect of ascorbic acid on microcirculation in patients with type II diabetes: A randomized placebo-controlled cross-over study. Clin Sci (Lond).

[62] Sankhla M., Sharma T.K., Mathur K., Rathor J.S., Butolia V., Gadhok A.K., Vardey S.K., Sinha M., Kaushik G.G. (2012). Relationship of oxidative stress with obesity and its role in obesity induced metabolic syndrome. Clin. Lab..

[63] De Roos N.M., Bots M.L., Schouten E.G., Katan M.B. (2003). Within-subject variability of flow-mediated vasodilation of the brachial artery in healthy men and women: Implications for experimental studies. Ultrasound Med. Biol..

