# Supplementary material for: Effects of Supplementation with the Fat-Soluble Vitamins E and D on Fasting Flow-Mediated Vasodilation in Adults: A Meta-Analysis of Randomized Controlled Trials
Source: Nutrients. 2015 Mar 10;7(3):1728–43. doi: 10.3390/nu7031728 (PMC4377878; doi:10.3390/nu7031728)
Supplement: Supplementary File 1 [file nutrients-07-01728-s001.docx]

Supplementary Information

**Figure S1.** Dose-response relationship between the dose of the vitamin E supplement and the change in fasting flow-mediated vasodilation (FMD). The size of each circle is proportional to the inverse of the variance of the change in FMD, and the line represents the regression line determined by meta-regression analysis. The dose of the vitamin E supplement was not associated with the change in FMD (*P* = 0.744).

**Figure S2.** Funnel plot for the effect of vitamin E supplementation on flow-mediated vasodilation (FMD) in random controlled trials (RCTs). Changes in FMD were plotted on the horizontal axis and the standard errors (s.e.) on the vertical axis. Visual evaluation of the funnel plot did not indicate presence of publication bias. The circle indicates the study with the most pronounced effect [1].

**Figure S3.** Funnel plot for the effect of vitamin D supplementation on flow-mediated vasodilation (FMD) in random controlled trials (RCTs). Changes in FMD were plotted on the horizontal axis and the standard errors (s.e.) on the vertical axis. Visual evaluation of the funnel plot did not indicate presence of publication bias.

Reference

1. Paolisso, G.; Tagliamonte, M.R.; Barbieri, M.; Zito, G.A.; Gambardella, A.; Varricchio, G.; Ragno, E.; Varricchio, M. Chronic vitamin E administration improves brachial reactivity and increases intracellular magnesium concentration in type II diabetic patients. *J. Clin. Endocrinol. Metab.* **2000**, *85*, 109–115.

© 2015 by the authors; licensee MDPI, Basel, Switzerland. This article is an open access article distributed under the terms and conditions of the Creative Commons Attribution license (http://creativecommons.org/licenses/by/4.0/).
